# Intraaortic Balloon Pump Counterpulsation and Cerebral Autoregulation: an observational study

**DOI:** 10.1186/1471-2253-10-3

**Published:** 2010-03-12

**Authors:** Judith Bellapart, Shureng Geng, Kimble Dunster, Daniel Timms, Adrian G Barnett, Rob Boots, John F Fraser

**Affiliations:** 1 Department of Intensive Care, Royal Brisbane and Women's Hospital, (Butterfield Street), Herston (4029), Australia; 2 Critical Care Research Group and Department of Intensive Care Medicine, The Prince Charles Hospital and University of Queensland, (Rode road), Brisbane, (4032), Australia; 3 Institute of Health and Biomedical Innovation & School of Public Health, Queensland University of Technology, (George Street), Brisbane, (4000), Australia

## Abstract

**Background:**

The use of Intra-aortic counterpulsation is a well established supportive therapy for patients in cardiac failure or after cardiac surgery. Blood pressure variations induced by counterpulsation are transmitted to the cerebral arteries, challenging cerebral autoregulatory mechanisms in order to maintain a stable cerebral blood flow. This study aims to assess the effects on cerebral autoregulation and variability of cerebral blood flow due to intra-aortic balloon pump and inflation ratio weaning.

**Methods:**

Cerebral blood flow was measured using transcranial Doppler, in a convenience sample of twenty patients requiring balloon counterpulsation for refractory cardiogenic shock (N = 7) or a single inotrope to maintain mean arterial pressure following an elective placement of an intra-aortic balloon pump for cardiac surgery (N = 13). Simultaneous blood pressure at the aortic root was recorded via the intra-aortic balloon pump. Cerebral blood flow velocities were recorded for six minute intervals at a 1:1 balloon inflation-ratio (*augmentation of all cardiac beats*) and during progressive reductions of the inflation-ratio to 1:3 (*augmentation of one every third cardiac beat*). Real time comparisons of peak cerebral blood flow velocities with systolic blood pressure were performed using cross-correlation analysis. The primary endpoint was assessment of cerebral autoregulation using the time delay between the peak signals for cerebral blood flow velocity and systolic blood pressure, according to established criteria. The variability of cerebral blood flow was also assessed using non-linear statistics.

**Results:**

During the 1:1 inflation-ratio, the mean time delay between aortic blood pressure and cerebral blood flow was -0.016 seconds (95% CI: -0.023,-0.011); during 1:3 inflation-ratio mean time delay was significantly longer at -0.010 seconds (95% CI: -0.016, -0.004, P < 0.0001). Finally, upon return to a 1:1 inflation-ratio, time delays recovered to those measured at baseline. During inflation-ratio reduction, cerebral blood flow irregularities reduced over time, whilst cerebral blood flow variability at end-diastole decreased in patients with cardiogenic shock.

**Conclusions:**

Weaning counterpulsation from *1:1 to 1:3 inflation ratio *leads to a progressive reduction in time delays between systolic blood pressure and peak cerebral blood flow velocities suggesting that although preserved, there is a significant delay in the establishment of cerebral autoregulatory mechanisms. In addition, cerebral blood flow irregularities (*i.e. surrogate of flow adaptability*) decrease and a loss of cerebral blood flow chaotic pattern occurs during the end-diastolic phase of each beat in patients with cardiogenic shock.

## Background

Aortic counterpulsation reduces myocardial ischemia by decreasing left ventricular afterload and increasing coronary blood flow. Pulse pressure variation is modified by the increase of diastolic-pressure time index and tension time index ratio (DPTI/TTI ratio). These principles are the physiological basis upon which the intra-aortic balloon pump (IABP) is used in states of cardiogenic shock and placed peri-cardiac surgery [1,2]. Although some studies have shown that IABP also increases cerebral blood flow (CBF) in patients with subarachnoid hemorrhage and cerebral vasospasm [3], other studies have reported reductions in CBF, specifically inducing a phenomenon of transient reversed diastolic flow with secondary reversed diastolic cerebral blood flow velocity (CBFV) [4]. Cheung and colleagues demonstrated that IABP modifies the CBFV waveform, mirroring that of BP without affecting the mean CBFV [5]. Acute reductions in CBFV at end-diastole can be compensated by an increase in mean CBFV [5]. However, Cheung and colleagues assessed CBF in clinically stable patients requiring prophylactic insertion of IABP, attributing the stability of the mean CBFV to a presumed intact cerebral autoregulation, but their study did not assess cerebral autoregulation.

Cerebral autoregulation is the mechanism by which constant CBF is maintained despite changes in cerebral perfusion pressure (CPP). Cerebral autoregulation mediates states of hyperemia or ischemia to avoid vasogenic edema or infarction respectively [6]. Impaired autoregulation has been regarded as a risk factor associated with adverse neurological outcome after cardiac surgery [7,8], head injury [9-11] and subarachnoid hemorrhage [12]. Cerebral autoregulation responds to spontaneous and induced changes in arterial blood pressure (BP) such as those occurring with IABP [13-15]. Neurological complications such as dense hemiplegia and long-term cognitive impairment are the most devastating adverse events after cardiac surgery [16], being these, mediated by an impaired cerebral autoregulation particularly in the elderly [7,8] and during reperfusion processes [17]. During periods of global hypoperfusion, certain regions within the brain are more susceptible to developing an ischemic penumbra [18-20]. It is in these areas where cerebral autoregulation may be most impaired, aggravating the primary tissue ischemia [21]. Cerebral autoregulation has been extensively studied using transcranial Doppler (TCD) which measures CBFV as a surrogate of CBF [22-25]. From all these methods, cross-correlation function (CCF) incorporates powerful computerized systems that enable the analysis of the phase shifts between BP and CBFV waveforms with the advantage of being useful for continuous testing of cerebral autoregulation at the bedside [26,27]. Using cross-correlation function, cerebral autoregulation is defined as preserved when there is a time delay up to 2 seconds and a negative correlation between BP and CBFV [27].

Whilst the use of IABP has represented a significant advantage in cardiovascular support and is often a necessary adjuvant for myocardial recovery, it is yet not clear what repercussions in CBF may imply, specifically during the weaning phase. This is the first study to assess cerebral autoregulation in a population at high risk of postoperative neurological damage during weaning of counterpulsation therapy.

## Methods

As an exploration of concept, a sample of 20 patients was recruited from two intensive care units. Group one comprised 13 patients requiring an elective insertion of an IABP following angioplasty or cardiac surgery and only one vasopressor to maintain mean arterial pressure (MAP). Group two consisted of seven patients with cardiogenic shock requiring an IABP as well as two or more vasopressors for MAP support, a persistent lactic acidosis and evidence of multiple-organ failure. Vasopressors were chosen at the clinician's discretion. The heterogeneous group size was not a limitation as intergroup comparisons were not the aim of the study. All patients recruited in this study gave informed consent.

A total of ten patients had undergone coronary artery bypass surgery (CABG); ten had been treated with pharmacological measures and coronary angioplasty. The proportion of CAGB patients was balanced between the two groups. All patients were in the acute phase of their illness (Table 1).

**Table 1:** Clinical characteristics of the study sample

Patient Group	Procedure	Vasopressor	CVP (mmHg)	Baseline PC02 mmHg	mean BPsystolic (1 to 1 inflation ratio) and SD in mmHg	Mean BPsystolic (1 to 2 inflation) ratio and SD, in mmHg	Mean BP systolic (1 to 3 inflation ratio) and SD, in mmHg	Diastolic Reversal	Troponin (ug/L)	LV. EF
1	MI + angioplasty	DBT	10	41	86 (2.9)	91.4 (1)			N/A	
1	MI + Lyses	DBT	9	38	106.4 (4.8)	106.8 (3)	105.9 (5.1)		N/A	20%
1	MI + lyses	DPM	9	40	134.4 (3.1)	142.4 (2.6)			0 9	20%
1	MI+ angioplasty	DPM	8	42	83.3 (8.4)	88.9 (5.4)	82.5 (6.4)		>100	20%
1	MI + CABG	DBT	14	36	111.2 (3)	113.9 (10)			>100	N/A
1	MI + angioplasty	DPM	8	38	148.5 (5.3)	147.4 (4.8)			6.9	35%
1	MI + CABG	DPM	14	40	125.9 (3.9)	131 (4)	129.9 (6.7)		> 50	30%
1	MI + Lyses	DPM	5	43	102.8 (1.9)	104.7 (4.4)			5	17%
1	MI + Angioplasty	DPM	9	40	156.3 (3.1)	164.2 (7.9)	162.1 (7.6)	yes	>100	N/A
1	MI + CABG	DPM	7	41	117 (4.2)	123.6 (5.6)	128.7 (3.3)	yes	4.6	68%
1	MI + CABG	DPM	14	43	105.1 (3.6)	102.9 (4.5)	102.3 (3.7)		38	>60%
1	MI + Angioplasty	DBT	22	40	78.9 (1.9)	100.7 (3.3)	107.5 (4.3)		37	>50%
1	MI + CABG	DPM	18	36	107.2 (4.9)	104.5 (4.5)	99.1 (3.1)		0.3	62%
2	MI + Lyses	DBT/NA/DPM	10	40	110.4 (5.5)	111.8 (5.9)	110.9 (5.9)		>100	N/A
2	MI + CABG	DBT/NA/DPM	17	36	116.6 (1.1)	118.5 (2.0)		yes	>100	N/A
2	MI + Lyses	ADR/NA/DPM	19	41	86.0 (2.3)	88.5 (1.8)			60	N/A
2	MI + CABG	/NA/ADR	12	35	94.7 (1.5)		103.5 (2.0)		>100	30%
2	MI + CABG	NA/ADR/VAS	9	37	98.1 (1.0)	101.9 (1.4)			5	20%
2	MI +CABG	NA/VAS/LEV	13	45	124.3 (4.3)	131.7 (12)	138.4 (4.9)		100	45%
2	MI + CABG	ADR/NA	12	44	98.5 (2)	105.6 (2.1)			4.2	N/A

CVP indicates central venous pressure; LV. EF, indicates left ventricular ejection fraction; DPM (Dopamine); DBT (Dobutamin); NA (Noradrenalin); VAS (Vasopressin); ADR (Adrenalin); LEV (Levosimendan); N/A indicates not available; SD, indicates standard deviation.

Patient Group 1 = patients with IABP but not in cardiogenic shock. Group 2 = patients with IABP and cardiogenic shock.

Maintenance of a stable mean arterial pressure during inflation ratio reduction was ensured to allow a stable cerebral perfusion pressure and minimize interference with cerebral autoregulation. Patients were used as their own controls. Two patients were removed from the study due to significant hypotension (pre-defined as a reduction in BP systolic of more than 20% of baseline) during reduction of counterpulsation ratio. Data for 1:3 counterpulsation ratio was only collected in 11 patients, as the permission to wean counterpulsation was based on clinician judgment.

CBFV of middle cerebral arteries (MCAs) were measured using TCD following referenced criteria at the temporal acoustic window [23,24]. Both MCAs were insonated and the side with best acoustic characteristics chosen for study. TCD machines (DWL^® ^Sipplingen, Germany) used 2-MHz probes. Intra-patient variability was minimized using recordings only collected by one investigator formally trained in TCD [25].

Blood pressure signals from the IABP output channel and CBFV signals from the TCD device were simultaneously recorded using Powerlab (AD technology, model ML880, New South Wales, Australia) to minimise errors and bias which would be present if peripheral arterial pressure had been transduced. Stability of the insonated vessel diameter was assumed by maintaining a stable partial pressure of arterial carbon dioxide (PaCO2) during measurements. This assumption has been used previously in situations where transcranial color coded technology (which enables direct visualization and measurement of vessels diameter), is not available [6]. Patient care, including cardiovascular parameter goals and sedation were at the discretion of the treating clinician. Midazolam with minimal aliquots of fentanyl were used for analgesia and sedation in all cases. Propofol™ was not used due to the poor cardiac function and precarious haemodynamics in all these patients.

**Figure 1:**
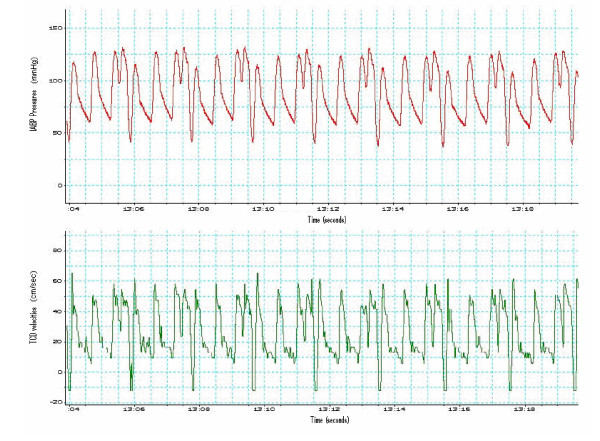
**Aortic pressures and Transcranial Doppler tracings**. Upper figure: IABP pressures (*y axis*) during 1:3 inflation-ratio over time (*x axis*). IABP augments every third cardiac beat, showing characteristic traces such as an augmented peak diastole, reduced end-diastolic pressure and decreased non-augmented peak systole; these pulse pressure variations imply a left ventricular afterload reduction whilst increasing diastolic pressures (*to improve coronary perfusion*). Lower figure: Cerebral blood flow velocities using TCD (*y axis*) at the Mean Cerebral artery, over time (*x axis*). Notice that the end-diastolic velocities become negative; known as "reversal flow" and are considered to be the result of a steeling phenomena from the deflation of the IABP in the Aortic root. Both traces are represented in a real time, beat-to-beat correlation, using the integrated software of a Power lab computer.

An initial three minute steady-state CBFV was recorded. With the IABP at a ratio of 1:1 a total of six minutes was recorded. Subsequently, the IABP timing was reduced to 1:2 and a further six minute recording of CBFV was taken to ensure the establishment of autoregulatory changes. This data collection was then repeated using a counterpulsation ratio of 1:3. After completion of the 1:3 ratio recordings (Figure 1), counterpulsation was returned to a 1:1 ratio and a further six minute recording collected.

Institutional Ethics Committee approval for the performance of the study was granted. All patients or their next of kin gave informed consent prior to enrolment in the study.

### Data analysis

Cerebral autoregulation was assessed using both linear and non-linear analysis. The former was based on pre-filtered data and application of the cross-correlation statistic. Cross-correlation measures the time between the peak of the CBFV at the level of the MCA and the peak of BP for each heart beat in real time. Several authors have found time delays between BP and CBFV up to a 2 seconds in healthy subjects and delays near to zero when autoregulation is impaired. With loss of cerebral autoregulation, changes in BP are directly transmitted to CBF without any delay in time. Conversely, when autoregulation is intact, buffering mechanisms within the cerebral vessels take place to accommodate BP changes without disturbing CBF dynamics, a "time consuming" process. By giving a trend rather than an absolute value a more realistic description of cerebral autoregulation is achieved rather than a dichotomous description of preserved/non-preserved autoregulation. This test has been extensively used and validated for the assessment of cerebral autoregulation both in dynamic and static conditions [26,27].

For the non-linear analysis we used a third-order moment test as previously described [28]. The cross-correlation is a second order test. By examining two points in time it is able to measure only the linear delay in time between the two signals. A test in the third-order, examines three points in time, and so is able to detect more complex (*non-linear*) patterns. It is also able to detect non-stationary or chaotic signals (*signals whose mathematical properties change randomly with time*).

For the cross-correlation analysis, BP and MCA velocity signals were bandpass filtered using a fifth-order Butterworth filter, into three frequency ranges: very low (0.015-0.07 Hz), low-frequency (0.07-0.15 Hz) and high-frequency (0.15-0.40 Hz) before applying cross-correlation [26]. The time series were normalized so that the autocorrelations at lag zero were one. The peak in the cross-correlation was used to estimate the average delay in seconds (using the *xcorr *function in Matlab signal processing toolbox). The time delay was estimated for every subject and IABP ratio combination.

Time delays were virtually identical for all three frequency ranges. As such, subsequent analysis was performed on the very low frequency range only. No other data pre-processing was applied [26]. Other researchers have applied windowing (*sampling by seconds*) to further filter data; however, in this study signals were at risk of being dampened (or even lost) using windowing. Therefore, to minimize the risk of missing important signals, data was kept in a raw form at the expense of some increased noise.

**Figure 2:**
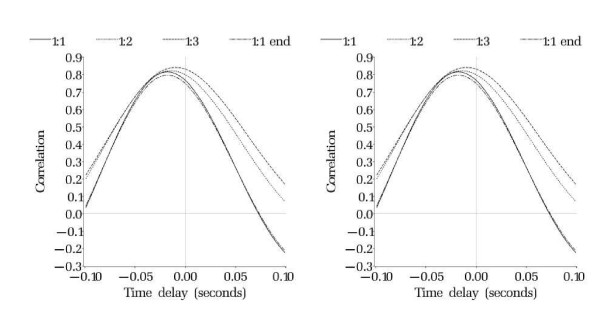
**Time delays and inflation ratios**. Time delays in couples of group 1 - left and group 2 - right, showing the relative delay for 1:1(continuous line), 1:2 (dotted line), 1:3 (dashed line) and 1:1-end (intermittent dashed-dotted line) IABP inflation ratio.

Patterns in time delays among subjects and by IABP ratio were examined using multiple regression modeling with IABP ratio and group as independent variables. A mixed model with a random intercept for each subject was used to control for repeated within-subject measurements [29]. This model was fitted using *Proc Mixed *in *SAS*. To give a graphical picture of the overall delay, the average cross-correlation for each balloon and cohort combination was plotted (Figure 2).

### Non-linear analysis

Non-linear differences were assessed using a test based in the third-order moment [28]. Initially, series were combined by dividing the IABP by MCA velocity and then log-transformation to give a series with more stable variance. Non-linearities were also assessed in the time domain from zero to 0.1 seconds. Separate tests for each subject and IABP ratio combination were performed and the results combined by overlaying each subject's significant region of non-linearity for the same IABP ratio to identify the times of common non-linear interactions. Regions of non-linear interactions where then plotted for each IABP ratio. Third-order non-linearity was calculated using the "bootstrap" function (Mathworks Australia Pty Ltd).

### Beat-to-beat analysis

To visually understand the changes in the IABP and MCA velocity with decreasing balloon ratio, mean time series per beat were plotted (the mean of the log-transformed ratio of IABP to MCA velocity). Each subject's beat length was initially estimated and the mean IABP and MCA velocity subtracted within each beat "window". The first 100 beats were then averaged from the start to the end of the beat in 0.001 second gaps. Mean beats for two subjects (each subject belonging to each study group) were plotted. This was a graphical analysis with no formal statistical testing.

## Results

Phase delays between peak CBFV and systolic IABP at the very low frequency range, decreased with a reduction in counterpulsation ratio with mean delays for 1:1, 1:2 and 1:3 counterpulsation ratios of -0.016 seconds (95% CI: -0.023, -0.011), -0.013 seconds (95% CI: -0.019, -0.007) and -0.010 seconds (95% CI: -0.016, -0.004) respectively; 1:1 counterpulsation ratio compared to 1:2, P < 0.0001; 1:1 counterpulsation ratio compared to 1:3, P < 0.0001. Following return to a 1:1 counterpulsation ratio, the phase delay increased between peak CBFV and systolic BP (-0.017 seconds, 95% CI: -0.023 -0.011) being unchanged from initial 1:1 measures (P = 0.62, Table 2). High correlation coefficients were found between counterpulsation ratio and phase delays (Table 3). There were no phase delay differences between patients with cardiogenic shock and those receiving prophylactic counterpulsation.

**Table 2:** Time delays for IABP ratios Time delay (seconds)

*Balloon ratio*	*Mean Time delay*	*95%*	*CI*	*t-value*	p-value*
1:1	-0.01664	-0.02253	-0.01076	-5.70	<.0001
1:2	-0.01274	-0.01862	-0.00685	-4.37	<.0001
1:3	-0.01011	-0.01620	-0.00403	-3.35	0.0017
1:1 end	-0.01691	-0.02280	-0.01102	-5.79	<.0001

CI: confidence interval

* Testing the null hypothesis that the time delay is zero

**Table 3:** Cross-correlation coefficients for time delays and groups

group	time	N	Mean	Min	Max
group 1	1-to-1	13	0.83	0.71	0.94
	1-to-2	13	0.84	0.74	0.94
	1-to-3	7	0.86	0.80	0.89
	1-to-1 end	13	0.82	0.72	0.89
group 2	1-to-1	7	0.88	0.81	0.93
	1-to-2	7	0.88	0.83	0.92
	1-to-3	3	0.91	0.90	0.93
	1-to-1 end	7	0.87	0.78	0.94

**Figure 3:**
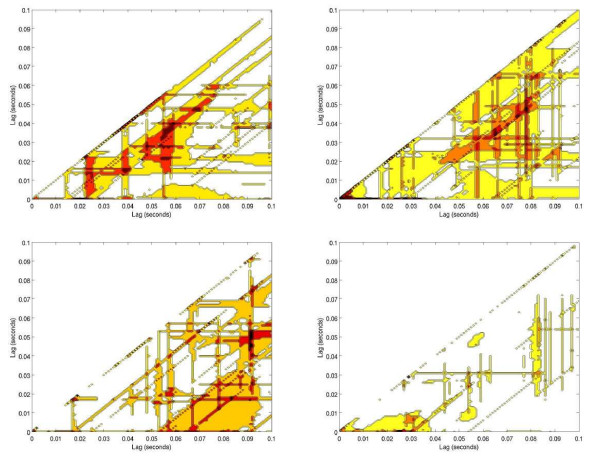
**Cerebral blood flow variability represented in a scale of colors**. Non-linearities represented in dark orange colors, shifting to the right on the (x) axis, for group 1(patients electively supported with IABP): Top-left: 1:1 ratio; top-right: 1:2 ratio; bottom left: 1:3 ratio; bottom right: 1:1-end ratio. Notice the progressive increase of time-lag of the cerebral blood flow variability as the IABP inflation-ratio is weaned; suggesting that cerebral blood flow variability takes longer to be established as the IABP is weaned off.

**Figure 4:**
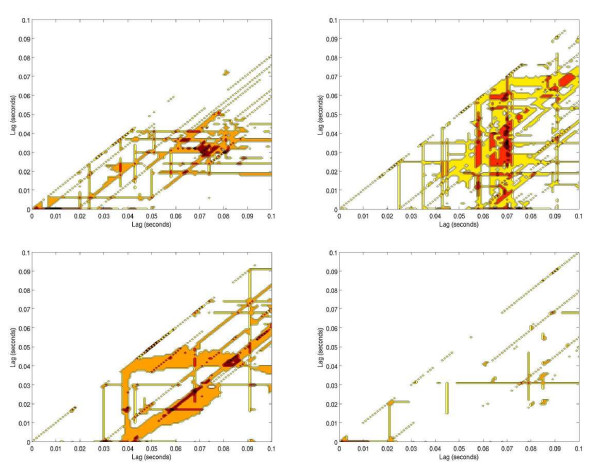
**Cerebral blood flow variability represented in a scale of colours**. Non-linearities represented in dark orange colours, shifting to the right on the (x) axis, for group 2 (patients in refractory shock): Top-left: 1:1 ratio; top-right: 1:2 ratio; bottom left: 1:3 ratio; bottom right: 1:1-end ratio. Notice the progressive increase of time-lag of the cerebral blood flow variability as the IABP inflation-ratio is weaned; suggesting that cerebral blood flow variability takes longer to be established as the IABP is weaned off. Overall reduction in cerebral blood flow variabilities are shown, when compared with subjects in group 1-fig 3.

Non-linearities in the ratio of IABP to MCA velocity within lags of 0.1 seconds were noted. As the counterpulsation ratio decreased from 1:1 to 1:3, the common region of non-linearity showed longer time delays demonstrated by areas of the common regions shifting from left to right along the x-axes (Figures 3, 4).

**Figure 5:**
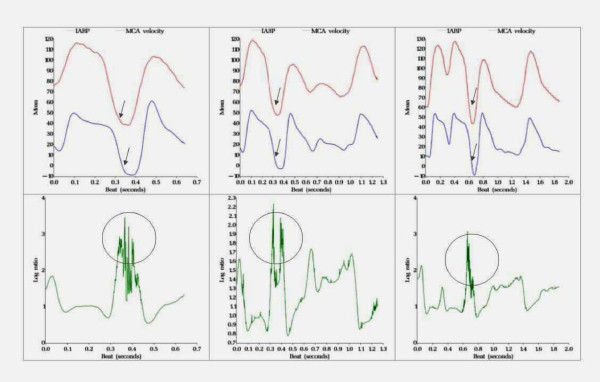
**Representation of cerebral blood flow chaotic pattern**. Real tracing from a representative subject in group 1 (*patients with elective support of IABP*) showing: Upper row: left figure corresponds to 1:1 IABP inflation-ratio trace (*in red*) superimposed to CBFV (*in blue*) during the same beat. Middle figure corresponds to 1:2 IABP inflation-ratio trace (*in red*) superimposed to CBFV (*in blue*) during another beat. Right figure corresponds to IABP inflation-ratio trace (*in red*) superimposed to CBFV (*in blue*) during a cardiac beat. Lower row: simultaneous representation of CBFV non-linearities (*variability*) expressed as a time function of log ratio (*y - axis*) and time (*x - axis*). Notice that the moment of maximum variability corresponds to the end-diastole in each beat

**Figure 6:**
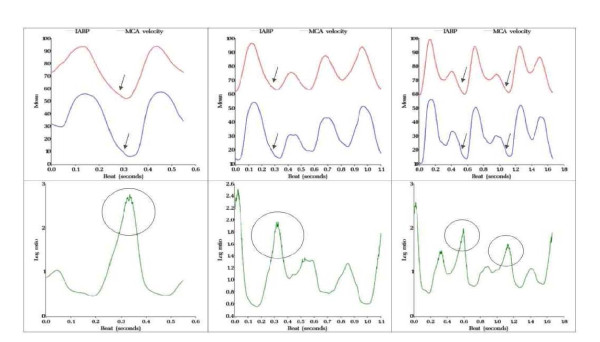
**Representation of cerebral blood flow chaotic pattern**. Real tracing from a representative subject in group 2 (*patients in refractory shock*) showing: Upper row: left figure corresponds to 1:1 IABP inflation-ratio trace (*in red*) superimposed to CBFV (*in blue*) during the same beat. Middle figure corresponds to 1:2 IABP inflation-ratio trace (*in red*) superimposed to CBFV (*in blue*) during another beat. Right figure corresponds to IABP inflation-ratio trace (*in red*) superimposed to CBFV (*in blue*) during a cardiac beat. Lower row: simultaneous representation of CBFV non-linearities (*variability*) expressed as a time function of log ratio (*y - axis*) and time (*x - axis*). Notice that the moment of maximum variability corresponds to the end-diastole in each beat, however in this case variability is minimum when compared with group 1.

The beat-to-beat plots show the mean of the first 100 beats for a patient in groups one (Figure 5) and two (Figure 6). The log-ratio of the two series was always greatest at the area of the IABP end-diastole indicating that the greatest relative difference in the two series occurred at these areas. It is in these areas where the group one subject displays chaotic behavior, as the pattern becomes more erratic when compared with the smoother pattern seen in group two.

## Discussion

This study demonstrates a significant decrease in the phase delay between systolic IABP and peak CBFV as counterpulsation inflation ratio is reduced. In addition, time delays are negative indicating that the changes in CBFV precede those of the systemic arterial tree recorded by the IABP, a behavior previously reported by other authors indicating that cerebral autoregulation is preserved [27]. The progressive shortening of time delays suggest that although preserved, cerebral autoregulation shows a loss of efficacy through the phases of inflation ratio reduction. This pattern differs from the dichotomous description of cerebral autoregulation, i.e. an "*all or nothing" *state of cerebral autoregulation and represents an assessment in agreement with other authors [26]. Finally, high correlation coefficients found between inflation ratios and time delays of systolic pressures and peak cerebral flow velocities, indicate that there is a changeable autoregulatory response among ratio reduction phases.

Phase delays returned to baseline levels when the counterpulsation ratio returned to a 1:1 ratio, suggesting that there is an immediate recovery of the efficiency of autoregulation. Although the exact reason for this behavior is unclear, two explanations are plausible: Firstly, although not apparent in our study, non-significant reductions in MAP could have accompanied the change of IABP from 1:2 to 1:3, leading to small reductions in CBF which could have then diminished the efficiency of the autoregulatory response. Secondly, the reduction in the frequency of the counterpulsation balloon and therefore the lesser homogenous and more disturbed blood flow pattern exhausts the efficacy (*or latency in the response*) of cerebral autoregulation. Cheung and colleagues found that CBFV-to-pressure ratio at end-diastole was dependent on the magnitude of augmentation rather than the timing of inflation for both augmented and non-augmented beats [5]. However, early IABP deflation time leads to end-diastolic reversal of CBF [30]. This reversal flow pattern is magnified in patients with left ventricle dysfunction. In this setting of delayed isovolumetric contraction, IABP deflation occurring during a period when there is no blood ejection into the aorta, retrograde carotid artery blood flow occurs.

Although the use of different types of vasopressors could hypothetically interfere in the autoregulatory response, the pattern of progressive reduction in time delays with the reduction of inflation ratio, remain unchanged when compared with patients with minimal vasopressor requirements.

Currently, the two known modes of weaning IABP support are a reduction in ratio of augmented beats and a reduction of balloon inflation pressure while maintaining a support ratio of 1:1. There is no consensus as to which mode of weaning IABP support is superior. A strategy of weaning that avoids carotid flow reversal or impaired cerebral autoregulation could minimize neurological complications.

Cerebral autoregulatory efficiency deteriorates particularly in those patients with severe left ventricle dysfunction; with a reduction of counterpulsation ratio potentially risking cerebral hypoperfusion [7]. This study shows three patients with reversal end-diastolic pattern and a negative CBFV. This phenomenon requires further investigation to define if CBF reductions are related to IABP timing or to balloon augmentation pressure.

Time delays between peak CBFV and systolic IABP show a negative phase consistent with preservation of cerebral autoregulation. This "phase-led property" of cerebral autoregulation has been previously described with CBFV changes preceding BP changes [14].

Non-linear analysis shows that as IABP is weaned to 1:3 ratio, CBFV irregularities spread apart in time, shifting from left to right on the x axis (Figures 3, 4). An increase in chaotic activity at end-diastole was more common in the Group one subject when compared with a Group two subject (Figures 5, 6). This chaotic activity may reflect a more intact system. Several authors have reported beat-to-beat variability in CBF and CBFV [31,32]; perhaps the result of autonomic cerebral vasomotion [33] or oscillations in central control [34]. Variability in biological systems such as heart rate have been extensively studied and correlated with outcome [35,36]. Cerebral blood flow has been described as "multifractal" (*a behavior that is irregular, mathematically repetitive and explained by its own definition*) and chaotic in the normal population while demonstrating monofractal behavior when cerebral autoregulation is impaired [37].

### Limitations

This study focuses on the relationship between IABP pressures at the tip of the descending aorta *and CBFV *assessing their time delay using the cross-correlation approach [26,27,38]. This method has been previously used to assess the effect of spontaneous BP oscillations on CBFV in undisturbed systems. Although this study incorporated patients with pressure waveform modifications, these were continuously sustained over days, leading to a stable blood waveform pattern; for this reason, the authors considered that the cross-correlation method could be appropriate for the continuous monitoring of cerebral autoregulation. Similarly to previous studies, data stability was ensured by taking long recordings and allowing a three minute steady state-phase before definitive recording [26].

CBFV was initially recorded in both MCAs, but only the side with better acoustic properties was recorded and analyzed. Although spatial heterogeneity of cerebral autoregulation as well as interhemispheric differences are well described [39], an endpoint of this study was not to define interhemispheric differences, but rather to ensure the best transcranial Doppler recordings in order to minimize the signal-to-noise ratio and increase data reliability [40].

Many authors have found time delays within two seconds in healthy populations when compared with patient cohorts [14]. It could be argued that small differences in time delays, as shown in this study, may not be clinically relevant; however, the emphasis of this study lies upon the conceptual effect of IABP weaning using inflation ratio reduction in both, time delays and chaotic behavior of cerebral blood flow. In a population such as this, cerebral autoregulatory dysfunction has the potential for devastating consequences. Further studies comparing both counterpulsation weaning strategies (*inflation-ratio reduction and pressure reduction*) on cerebral autoregulation may be needed to define the implications of pulse pressure variation on cerebral autoregulation.

Although this study clinically defines two groups with presumed differences in left ventricular function, such separations may have been arbitrary as there were no autoregulation differences between patients in refractory cardiogenic shock and those receiving supportive counterpulsation.

Time domain cross-correlation functions are often applied to a series of signals in a different range of frequencies. Cerebral autoregulation is known to show high pass filter properties that enable low-frequency oscillations to be efficiently dampened by autoregulatory mechanisms while fluctuations in high-frequency ranges remain unfiltered [15]. Slow spontaneous oscillations occur at low frequencies in time series with continuous BP-CBFV recordings [41]. Our study processed data using bandpass filtering at three frequencies ranges prior to signal analysis, as previously described [42]. No differences in time delays were found among these frequency ranges and as such, results were presented using the very-low frequency filter only. Although the similarity among different frequency ranges has not been commonly reproduced, this finding could be explained by the lack of raw data smoothing, during the processing phase.

## Conclusions

This study shows that the use of fully augmented IABP does not adversely affect cerebral autoregulation. Reductions in inflation ratio during counterpulsation weaning progressively worsens cerebral autoregulation efficiency with the potential risk of cerebral hypoperfusion. Reduction in chaotic activity seen at end-diastole in patients with cardiogenic shock and inflation ratio reduction may also indicate impairment of CBF dynamics. Further studies must investigate optimal IABP weaning strategies to minimize the risk of reversed CBF at end-diastole, particularly amongst patients with poor residual left ventricular function.

## Abbreviations

IABP: Intra aortic balloon pump; CBFV: cerebral blood flow velocity; TCD: transcranial Doppler; BP: blood pressure; CBF: cerebral blood flow; DPTI/TTI: diastolic pressure time index and tension time index ratio; CPP: cerebral perfusion pressure; CCF: cross-correlation function; MAP: mean arterial pressure; CABG: coronary artery bypass graft; MCA: mean cerebral artery; PaC02: partial pressure of carbon dioxide.

## Competing interests

The authors declare that they have no competing interests.

## Authors' contributions

JB and JF conceived and designed the study; JB and SG undertook patient screen and data acquisition. JB drafted the manuscript which was then reviewed and amended by JF and RB. RB was involved in extensive editing. AB performed all data analysis and supervised the data interpretation. DT facilitated and advised about the use of the powerlab technology. KD resolved all technical problems related to data acquisition. All authors read and approved the final manuscript.

## Pre-publication history

The pre-publication history for this paper can be accessed here:

http://www.biomedcentral.com/1471-2253/10/3/prepub
